# Effects of Heat-Moisture-Treated High-Amylose Rice Flour on Body Weight, Lipid Metabolism, and Gut Microbiome Composition in Obese Rats

**DOI:** 10.3390/metabo13070858

**Published:** 2023-07-19

**Authors:** Sihui Ma, Sae Takasugi, Masayoshi Sugawara, Kenji Saito, Huijuan Jia, Hisanori Kato

**Affiliations:** 1 Health Nutrition, Graduate School of Agricultural and Life Sciences, University of Tokyo, Tokyo 113-0032, Japanskkj774@gmail.com (K.S.); 2 Department of Materials Engineering, National Institute of Technology, Nagaoka College, Niigata 940-0817, Japan; suga@nagaoka-ct.ac.jp

**Keywords:** heat-moisture treatment, amylose, lipid metabolism, bile acid, gut microbiome

## Abstract

The rising prevalence of lifestyle diseases, such as type 2 diabetes, cardiovascular diseases, and metabolic syndrome, has increased the need for effective dietary interventions. This study aimed to evaluate the effects of heat-moisture-treated high-amylose rice (HA-HMT) on body weight, lipid metabolism, and gut microbiome composition in a rat model of obesity. Starch digestibility—specifically, resistant starch—has been shown to provide various health benefits, including improved metabolic health and gut microbiome composition. We employed a sequential approach: firstly, utilizing diet-induced obesity rat models fed with HMT-processed and HMT-non-processed low- or high-amylose rice to investigate the potential of amylose content or HMT to alter phenotypic characteristics and lipid metabolism; and secondly, using the optimal rice flour identified in the previous step to explore the underlying mechanisms. Our findings indicate that heat-moisture treatment, rather than the level of the amylose content of the rice, contributes to the observed anti-obesity and cholesterol-lowering effects. We identified candidate genes contributing to the cholesterol-regulating potential and demonstrated that HMT rice flour could influence the gut microbiome, particularly the *Ruminococcus* taxa. This study provides valuable insights into the health benefits of HA-HMT rice and supports its potential as a functional food ingredient in the management of obesity and cholesterol-related disorders.

## 1. Introduction

The global prevalence of lifestyle diseases, including type 2 diabetes (T2D), cardiovascular diseases (CVD), and metabolic syndrome, is on the rise and has become the leading cause of death and disability [1]. Improving the nutritional quality of staple foods presents a promising complementary intervention strategy to reduce the burden of diet-related chronic diseases [2].

Starch is a major component of plant-based foods and the primary carbohydrate in human diets [3]. Absorption rate and glucose-releasing capacity are critical nutritional characteristics of starch. Based on these properties, starch can be classified into three categories: (1) rapidly digestible starch (RDS), which is primarily composed of amorphous starch and is quickly digested and absorbed in the upper small intestine, leading to rapid glycemic changes, with starch-to-glucose conversion (SGC) occurring within 20 min postprandially [4]; (2) slowly digestible starch (SDS), characterized by complex structures and slower digestion properties, featuring a postprandial SGC time of 20–100 min; and (3) resistant starch (RS), which is not converted to glucose for over 120 min after ingestion. Compared to RDS, SDS and RS result in a slower increase and decrease, respectively, in postprandial blood glucose levels as well as more stable hormone responses and glycemic index values [5]. RS, which is not readily digested in the small intestine, reaches the large intestine and serves as a substrate for fermentation by gut microbiota, which produce short-chain fatty acids that have beneficial effects on host metabolism and gut health [6]. These properties can lead to various health benefits, such as reduced blood pressure, cholesterol levels, and risks of T2D and CVD [7]. Specifically, changes in gut microbiota composition and function have been directly linked to obesity and other metabolic disorders. As such, modulation of the gut microbiota through dietary interventions, such as the consumption of high-amylose (HA) rice, could serve as an effective strategy for managing these conditions [8]. This study also aims to further elucidate this mechanism and establish the therapeutic potential of HA rice.

Rice (*Oryza sativa*) is a staple food and a significant source of carbohydrates in various cultures. During processing, the micronutrient-rich outer layers are often removed to improve taste. Rice grains that retain the bran and germ layers typically have a brown color and are, therefore, commonly referred to as brown rice (BR), distinct from the outer layer-ground, refined, white-colored rice. Whole grains, such as brown rice, may contribute to better glycemic control and reduced risk of type 2 diabetes when they replace refined grains and simple sugars in the diet, given that the latter are associated with increased risk of these conditions [9]. However, its starch digestibility largely depends on the processing technique employed.

Recognizing the nutraceutical importance of starch, efforts have been made to optimize its nutritional value through the development of novel processing techniques. Heat-moisture treatment (HMT) is a processing technique that involves the application of heat and moisture to starch-based foods, resulting in alterations to their physicochemical properties and, consequently, their potential health benefits [10]. HMT has been shown to increase the RS content in various starch-rich foods, including rice [11]. This contributes to stable postprandial blood glucose, insulin, non-esterified fatty acids, and cholesterol levels, further promoting metabolic health [12]. The research question we seek to address is how the absorption rate and glucose-releasing capacity, critical nutritional characteristics of starch, affect blood glucose levels and metabolic health, particularly in the context of alterations brought about by heat-moisture treatment.

Previous studies have investigated the impact of HMT on the functional properties of HA rice [13]; however, few have specifically addressed its effects on body weight, lipid metabolism, and gut microbiome in the context of obesity. Herein, we aimed to evaluate the influence of high-amylose heat-moisture-treated rice (HA-HMT) on body weight, lipid metabolism, and gut microbiome composition in a rat model of obesity.

The present study employed two approaches: the first utilized HMT-processed and HMT-non-processed low- or high-amylose rice and diet-induced obesity rat models to investigate the potential of amylose content or HMT to alter phenotypic characteristics and lipid metabolism; the second used the optimal rice flour identified previously to study the underlying mechanisms. Our findings will not only expand the current understanding of the nutritional and health benefits of HMT but also provide valuable insights for the development of effective dietary strategies to combat obesity and associated metabolic disorders.

## 2. Materials and Methods

### 2.1. Materials

The rice flour used for the studies was obtained from the National Institute of Technology. The fodder was made using different kinds of rice flour and other components. The nutritional information of the fodder is shown in Table 1 and Table 2. Rice flour that underwent HMT was treated in an oven set at 0.1 MPa/120 °C for 10 min.

### 2.2. Study Designs

#### 2.2.1. Study 1

**Figure 1:**
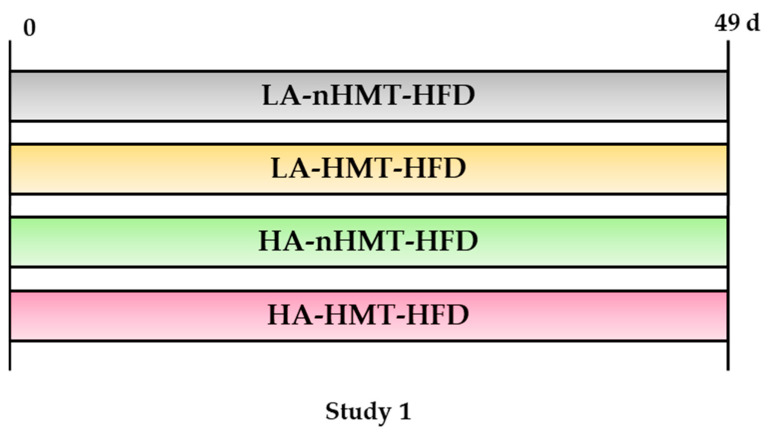
Design of Study 1. LA, low-amylose; HA, high-amylose; nHMT, without heat-moisture treatment; HMT, heat-moisture treatment; HFD, high-fat diet; LA-nHMT, low-amylose, high-fat diet without HMT; LA-HMT, low-amylose, high-fat diet with HMT; HA-nHMT, high-amylose, high-fat diet without HMT; HA-HMT, high-amylose, high-fat diet with HMT.

Twenty-nine male Wistar rats (5-week-old) were purchased from Charles River Japan (Kanagawa, Japan) and kept to cages (27 × 17 × 13 cm^3^) in a room maintained at 22 ± 2 °C with a relative humidity of 40–60% and a 12 h light (8:00–20:00) and 12 h dark (20:00–8:00) cycle. The animals were allowed free access to water and food throughout the experiment. After a 1-week adaptation, they were then randomly divided into four groups with the same average weight and four kinds of high-fat diets (HFDs): two diets whose carbohydrate source was rice flour obtained from low-amylose Koshihikari rice species, with or without HMT (LA-nHMT, *n* = 8; LA-HMT, *n* = 7); and two diets whose carbohydrate source was rice flour obtained from high-amylose Koshinokaori rice species, with or without HMT (HA-nHMT, *n* = 7; HA-HMT, *n* = 7). LA-nHMT, LA-HMT, HA-nHMT, and HA-HMT rice flour were obtained from the National Institute of Technology (Kosen), Nagaoka College (Nagaoka, Japan). The nutritional information of the diet is shown in Table 1. The study design is shown in Figure 1. All the diets in Study 1 were high-fat diets.

On the 49th day, the rats were anesthetized with isoflurane (Fujifilm Wako Pure Chemical Corp., Osaka, Japan) after a 16 h fasting. After euthanization, blood samples were collected from the carotid arteries. The liver, muscle, and abdominal fat pads were harvested and weighed. Plasma was separated from the blood, while the organs and tissues were snap-frozen in liquid nitrogen and stored at −80 °C until use.

#### 2.2.2. Study 2

**Figure 2:**
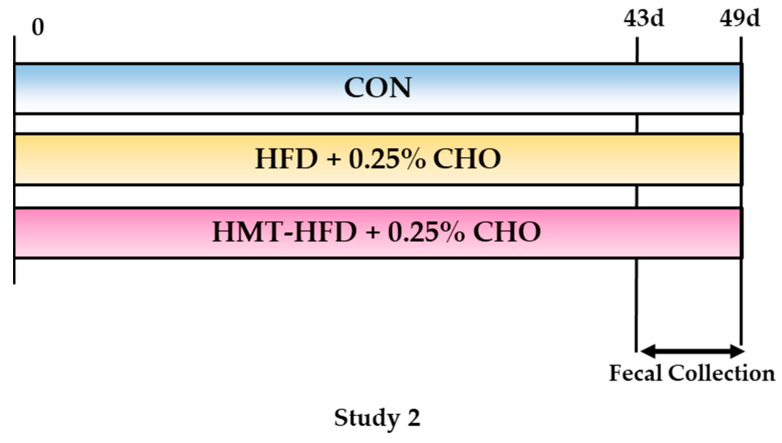
Design of Study 2. HMT, heat-moisture treatment; CON, control diet, without HMT; HFD, high-amylose, high-fat diet without HMT; HMT, high-amylose, high-fat diet with HMT; CHO, cholesterol. From the 44th to 49th day, rats were moved into metabolic cages, and from the 47th to 49th day, their feces were collected.

Study 2 was conducted to examine the triglyceride- and cholesterol-lowing effects we observed in study 1. Thirty male Wistar rats (5-week-old) were obtained and kept as described in Section 2.2.1. After a 1-week adaptation, the rats were then randomly divided into three groups with the same average weight with three kinds of diets: a control diet (CON, *n* = 10); HFD, whose carbohydrate source was rice flour obtained from low-amylose Koshihikari rice species, without HMT (HFD, *n* = 10); and HFD, whose carbohydrate source was rice flour obtained from high-amylose Koshinokaori rice species, with HMT (HMT, *n* = 10). For the HFD and HMT groups, extra cholesterol was added. Group 2 (HFD + 0.25% CHO) was used as a negative control of non-HMT processed diet, while CHO was supplemented. The nutritional information of the diet is shown in Table 2. From the 44th to 49th day, rats were moved into metabolic cages, and from the 47th to 49th day, their feces were collected. On the 49th day, blood and tissue were sampled by following the same procedures as documented in Section 2.2.1. The study design is shown in Figure 2.

### 2.3. Biochemical Tests

Blood and tissue biochemical parameters were measured according to the manufacturer’s protocol. Briefly, plasma biochemical parameters, including glucose, triglyceride (TG), total cholesterol (T-Cho), and high-density lipoprotein cholesterol (HDL-Cho) concentrations, were determined using commercial kits (Glucose C II test, Triglyceride E-test, and Cholesterol E-test, respectively; Wako Pure Chemical Industries, Osaka, Japan).

For hepatic TG and T-Cho, the lipid content was extracted from frozen livers using the modified Folch method [14]. Briefly, 100 mg of liver tissue was homogenized in 2 mL mixture of 2:1 (*v*/*v*) chloroform/methanol. The extracts were incubated overnight at 4 °C. Further, 0.4 mL of 0.8% KCI solution were added to each sample. After a series of vacuum evaporations, the organic phase was recovered using isopropanol. The recovered samples were tested for the presence of biochemicals. Hepatic TG and T-Cho levels were determined using commercial kits (Triglyceride E-test and Cholesterol E-test, respectively, Wako Pure Chemical Industries).

For fecal determination of bile acids, 50 mg of each fecal sample was dissolved in ethanol (0.5 mL) and kept for 2 h at 70 °C. After centrifugation for 5 min at 10,000 rpm, the supernatants were collected for subsequent analyses. Fecal bile acid was measured using a commercial kit (Total Bile Acid Test, Wako Pure Chemical Industries, Osaka, Japan).

### 2.4. RNA Extraction

Total RNA was isolated from homogenized liver tissue using NucleoSpinRNA (Takara Bio, Shiga, Japan), according to the manufacturer’s instructions. The total RNA concentration was measured using a NanoDrop spectrophotometer (ND-1000, NanoDrop, Wilmington, DE, USA). RNA quality was determined by assessing the A260/280 and A260/230 ratios and performing agarose gel electrophoresis.

### 2.5. DNA Microarray and Functional Analyses

Twelve rats from Study 2 (four from each treatment group), whose body weights were similar to the average weight of the respective group, were selected. DNA microarray analysis was performed using the Genechip Rat Genome 230 2.0 Array (Thermo Fisher Scientific, Waltham, MA, USA). Data were normalized by using the Robust Multi-array Average (RMA) method in R software, and differentially expressed genes between the groups were identified using the rank products 2.0 method. The probesets were filtered under the condition of a false discovery rate < 0.05. Bioinformatics analysis was performed using Ingenuity Pathway Analysis (IPA; QIAGEN, Inc., Germantown, MD, USA) to examine the underlying molecular mechanisms. IPA Core Analysis was used to identify the most significant biological canonical pathways in which the molecules in the dataset were involved.

### 2.6. Real-Time Quantitative Polymerase Chain Reaction (RT-qPCR)

cDNA was synthesized from the extracted total RNA using PrimeScript RT Master Mix (Takara Bio), according to the manufacturer’s instructions. RT-qPCR was performed using a Thermal Cycler Dice Real Time System TP800 (Takara Bio, Shiga, Japan). The expression level of ribosomal protein s18 (*Rps18*) was used as an internal reference for the normalization of target gene mRNA expression.

### 2.7. Metabolome Analysis

The plasma samples of nine rats from Study 2 (three from each treatment group), whose body weights were similar to the average weight of the respective groups, were selected for metabolome analyses. Briefly, samples were filtrated through 5 kDa cut-off filters (Ultrafree-MC-PLHCC, Human Metabolome Technologies, Yamagata, Japan) to remove macromolecules, and then resuspended in 50 μL of ultra-pure water using CE-TOFMS immediately before the metabolome analysis. The detected peaks were annotated based on the m/z value and normalized migration time. The relative area value of each peak was calculated and subsequently used for the intergroup comparison.

### 2.8. Cecal Bacterial DNA Extraction and Metagenome Analysis

QIAamp Stool Mini Kit (Qiagen, Hilden, Germany) was used to extract DNA from cecal contents, following the manufacturer’s instructions. The variable regions 3 and 4 of the 16S rRNA were amplified using the primers, 5′-CCTACGGGNGGCWGCAG-3′ and 5′-GACTACHVGGGTATCTAATCC-3′, followed by a modification to include the Illumina adapters and barcode sequences for the next sequencing. Library size and quantification analysis were conducted using the Agilent 2100 Bioanalyzer (Agilent Technologies, Santa Clara, CA, USA). All libraries were pooled in a single Illumina MiSeq run (MiSeq Reagent Kit V3, 600 cycles, Illumina, San Diego, CA, USA). The software of Quantitative Insights Into Microbial Ecology (QIIME v1.8.3) (http://www.qiime.org accessed on 20 May 2023) was used to analyze the data against the Greengenes alignment (v gg_13_8).

With a threshold of 97% pair-wise identity, we assigned the resulting sequences to operational taxonomic units and classified the representative sequences by the Ribosomal Database Project classifier in QIIME, following the Greengenes OUT database. A false discovery rate < 0.05 was considered significant.

### 2.9. Statistical Analysis

Results are expressed as the mean ± standard error of the mean (SEM). Multiple-group comparisons were performed using two-way (Study 1) and one-way (Study 2) analysis of variance (ANOVA), followed by Tukey’s test using the GraphPad Prism software (GraphPad Prism 9.0, San Diego, CA, USA). Statistical significance was set at a *p*-value of <0.05.

## 3. Results

### 3.1. Results of Study 1

#### 3.1.1. Food Intake and Body Weight Changes in Study 1

**Figure 3:**
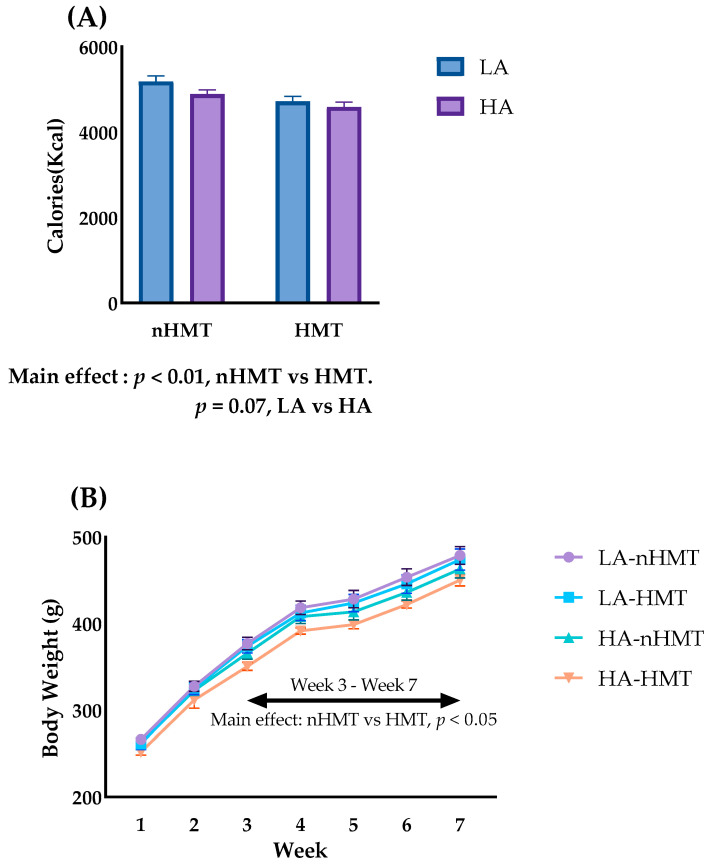
Changes in calorie intake and body weight in Study 1. (**A**) Total calorie intake; (**B**) Weight change throughout the experiment. Data are shown as means ± standard error of the mean (SEM), *n* = 7 or 8. LA, low-amylose; HA, high-amylose; nHMT, without heat-moisture treatment; HMT, heat-moisture treatment.

Figure 3 shows the total calorie intake and weight changes in rats throughout Study 1. As shown in Figure 3A, a significant main effect was found between the HMT and nHMT groups. A similar significant main effect was found on body weight change from the third week after the study began (Figure 3B). These results indicate that HMT reduces total calorie intake, and the decrease in food consumption may be one reason why rats in the HMT groups had decreased body weight. In contrast, amylose content did not show marked effects on total calorie intake or body weight change.

#### 3.1.2. Final Body Weight, Organ and Tissue Masses, and Blood and Tissue Biochemical Parameters in Study 1

After euthanization, the final body weight, organ, or tissue mass was recorded, and the ratio of organ or tissue mass/final body weight was calculated. As shown in Table 3, HMT had the main effect of reducing the final body weight. No significant effects of HMT and amylose content were observed on liver mass, adipose tissue mass, skeletal muscle mass, liver mass/body weight, adipose mass/body weight, and skeletal muscle/body weight. The plasma concentrations of TG, Cho, LDL-Cho, and HDL-Cho, and hepatic concentrations of TG were not altered by any factor. However, HMT showed a main effect of reducing the hepatic concentration of T-Cho. The results of Study 1 indicate the possible role of HMT in weight loss and its interaction with hepatic lipid metabolism; therefore, we conducted Study 2 based on these results.

### 3.2. Results of Study 2

#### 3.2.1. Food Intake and Body Weight Changes in Study 2

**Figure 4:**
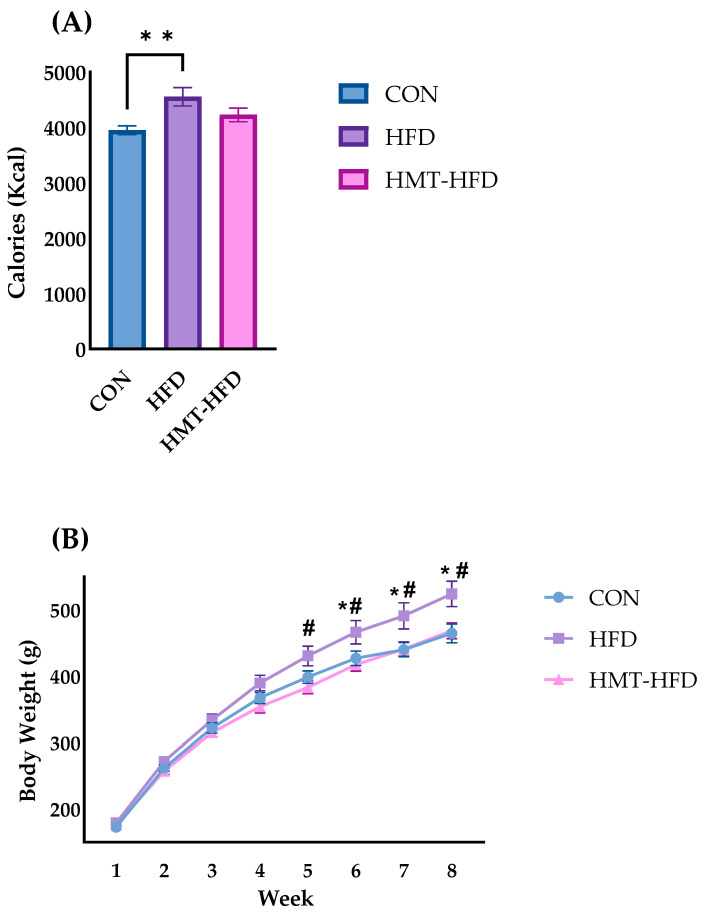
Changes in calorie intake and body weight of Study 2. (**A**) Total calorie intake; (**B**) Weight change throughout the experiment. Data are shown as means ± standard error of the mean (SEM), *n* = 9 or 10. CON, control diet; HFD, high-fat diet; HMT, heat-moisture treatment. * *p* < 0.05 and ** *p* < 0.01 vs. CON. ^#^ *p* < 0.05 vs. HMT.

Figure 4 shows the total calorie intake and weight changes in rats throughout Study 2. As shown in Figure 4A, the HFD group rats consumed significantly more total calories, while no significant difference was found in the HMT group rats. Rats in the HFD group exhibited significantly higher body weight than those in the CON or HMT groups, from week 5 and week 6, respectively (Figure 4B). At the end of Study 2, rats in the HMT group presented body weights nearly equal to those in the CON group, strongly implying that HMT may benefit the metabolic properties of the rice flour, leading to a resistance to weight gain, in the context of HFD administration.

#### 3.2.2. Final Body Weight, Organ and Tissue Masses, and Blood and Tissue Biochemical Parameters in Study 2

After euthanization, the final body weight and organ and tissue masses were recorded, and the ratio of organ or tissue mass/final body weight was calculated. As shown in Table 4, HFD significantly increased final body weight, liver mass, total adipose tissue mass, and the ratios of liver mass/body weight and adipose tissue mass/body weight (*p* = 0.05), compared to CON. However, despite the fact that the ratio of liver mass/body weight in the HMT group remained significantly higher than that in the CON group, HMT ameliorated the abnormal increase in total adipose tissue mass and the ratio of adipose tissue mass/body weight caused by HFD. Furthermore, as shown by the weight change curve presented in Figure 3B, rats in both the CON and HMT groups had distinctly lower final body mass than those in the HFD group.

#### 3.2.3. Biomarkers of Lipid Metabolism

**Figure 5:**
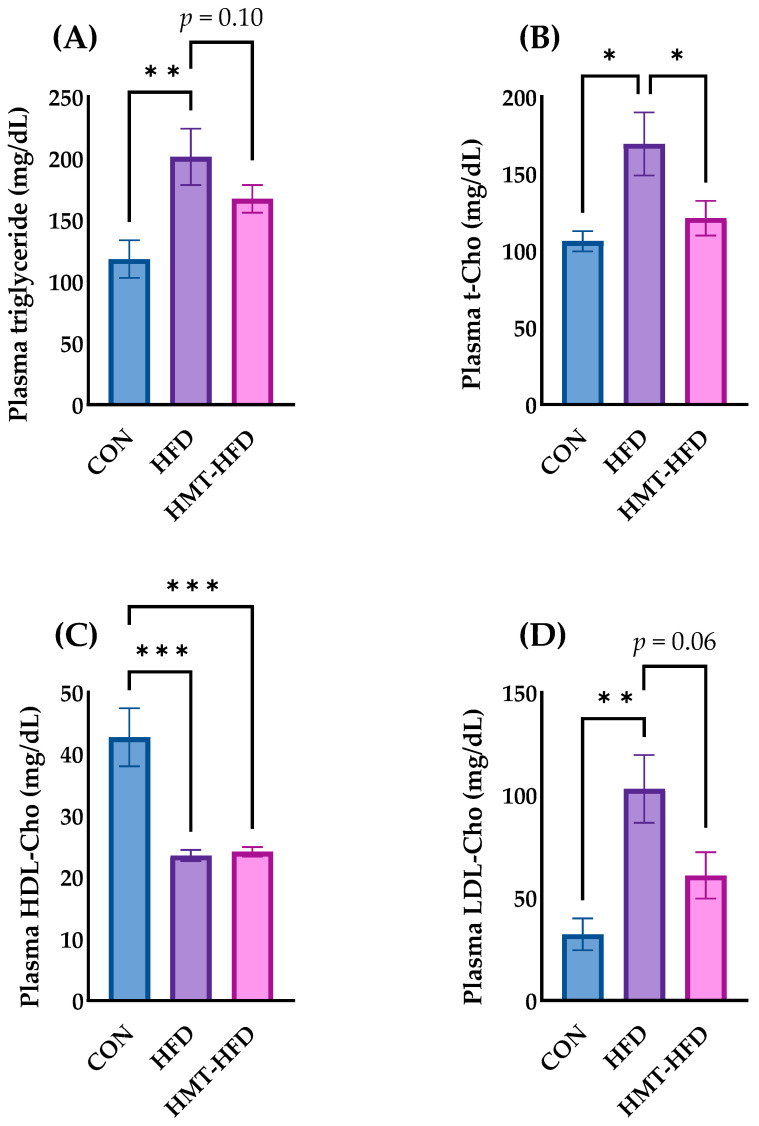
Changes in plasma cholesterol metabolites. (**A**) Plasma triglyceride; (**B**) Plasma total cholesterol; (**C**) Plasma high-density lipoprotein cholesterol; (**D**) Plasma low-density lipoprotein cholesterol. Data are shown as means ± standard error of the mean (SEM), *n* = 8–10. CON, control diet; HFD, high-fat diet; HMT, heat-moisture treatment; t-CHO, total cholesterol; HDL-Cho, high-density lipoprotein cholesterol; LDL-Cho, low-density lipoprotein cholesterol. * *p* < 0.05, ** *p* < 0.01, and *** *p* < 0.001 between the indicated groups.

Subsequently, the levels of the metabolites related to lipid metabolism were measured. As shown in Figure 5, HFD significantly elevated the plasma concentrations of TG (Figure 5A), T-Cho (Figure 5B), and LDL-Cho (Figure 5D) and significantly (*p* < 0.001) reduced the plasma concentration of HDL-Cho (Figure 5C). Interestingly, despite HMT having the same lipid content as HFD, rats in the HMT group had significantly lower concentrations of plasma T-Cho (Figure 5B), TG (*p* = 0.10, Figure 5A), and LDL-Cho (*p* = 0.06, Figure 5D). In contrast, HDL-Cho, which is considered to be the beneficial cholesterol, was decreased in both HFD and HMT groups (Figure 5C).

**Figure 6:**
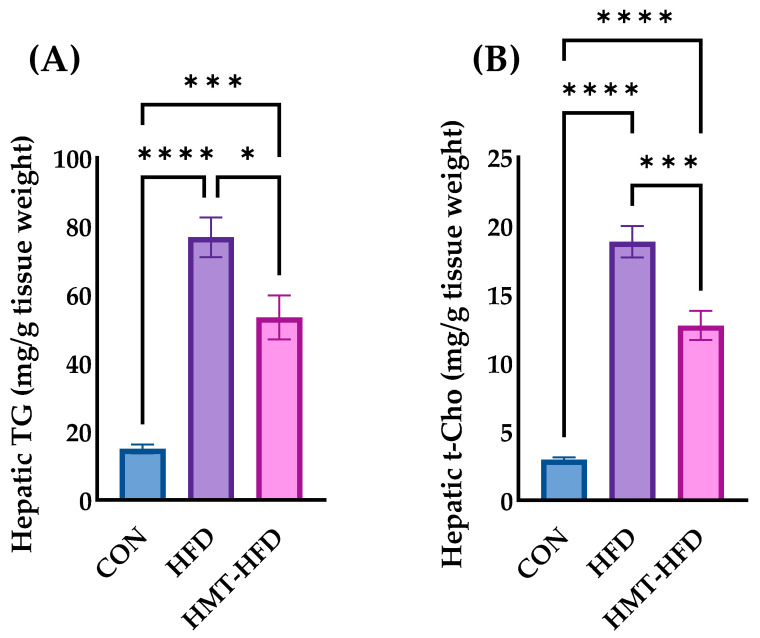
Changes in hepatic lipid metabolites. (**A**) Hepatic triglyceride; (**B**) Hepatic total cholesterol. Data are shown as means ± standard error of the mean (SEM), *n* = 9 or 10. CON, control diet; HFD, high-fat diet; HMT, heat-moisture treatment; TG, triglyceride; t-Cho, total cholesterol. * *p* < 0.05, *** *p* < 0.001, and **** *p* < 0.0001 between the indicated groups.

**Figure 7:**
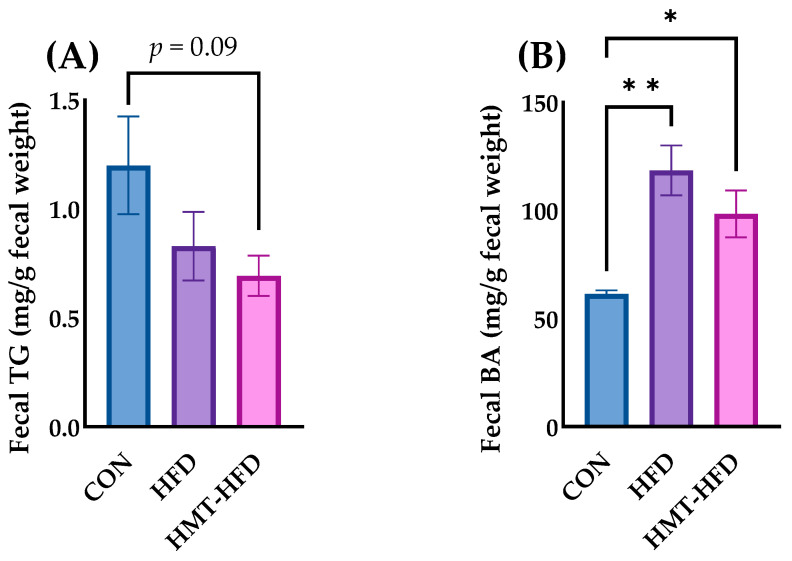
Changes in the levels of fecal (**A**) triglyceride and (**B**) bile acids. Data are shown as means ± standard error of the mean (SEM), *n* = 8 or 9. CON, control diet; HFD, high-fat diet; HMT, heat-moisture treatment; TG, triglyceride; BA, bile acids. * *p* < 0.05 and ** *p* < 0.01 between the indicated groups.

In the liver, TG (Figure 6A) and T-Cho (Figure 6B)showed the same behavior as their plasma counterparts (Figure 5A,B, respectively). HFD elevated both the hepatic TG and T-Cho levels, whereas HMT ameliorated these elevations. We also measured fecal TG and bile acid levels (bile acid is the final metabolite of cholesterol). As shown in Figure 7A, no significant differences in fecal TG levels were found among the groups. As depicted in Figure 7B, fecal bile acid was significantly elevated in both HFD and HMT groups, although the concentration was reduced in the HMT group compared to that in the HFD group.

We also conducted metabolome analyses using plasma samples (Table 5). The campesterol/cholesterol ratio was significantly lower in the HFD group than in the CON group. Although HMT alleviated this increase (HMT vs. HFD, *p* = 0.004, Table 5), the ratio in the HMT group remained lower than that in the CON group (HMT vs. CON, *p* = 0.019, Table 5). In contrast, the ratio of lathosterol/cholesterol was elevated in the HFD group only (HFD vs. CON, *p* < 0.019). As campesterol can compete with cholesterol for absorption and lathosterol is a cholesterol precursor, the above results indicate that less absorption and precursor might partially explain why rats in the HMT group had low circulating and hepatic cholesterol levels.

#### 3.2.4. DNA Microarray Results, IPA Pathway Enrichment Analyses Based on Transcriptome Results in Liver, and RT-PCR

**Figure 8:**
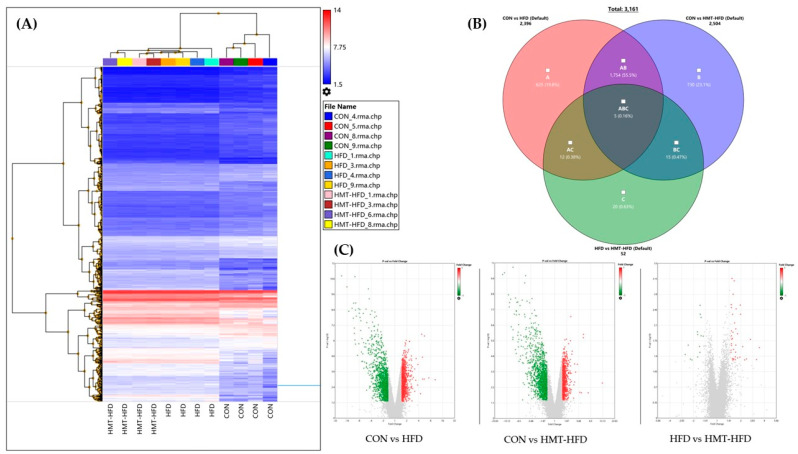
Transcriptome signatures. (**A**) Heat maps of average gene expression for each sample; (**B**) Venn diagram showing genes between groups that were differentially expressed (fold change ≥ 1.5) among groups; (**C**) Volcano plots visualizing log-fold changes in gene expression (x−axis) in relation to the negative logarithm of the adjusted *p*−value (y−axis) of the CON vs. HFD, CON vs. HMT, and HFD vs. HMT transcriptomic comparisons. HFD, high-fat diet; HMT, heat-moisture treatment. * *p* < 0.05 and ** *p* < 0.01 between the indicated groups.

Figure 8 shows the transcriptomic changes in the groups. Figure 7A shows general changes in the heat map. The results of the DNA microarray showed that 2396, 2504, and 52 genes were altered in the CON vs. HFD, CON vs. HMT, and HFD vs. HMT groups, respectively (Figure 8B). Figure 7C shows the visualized log-fold changes in gene expression (x-axis) in relation to the negative logarithm of the adjusted *p*-value (y-axis) using volcano plots. Employing QIAGEN IPA software, we focused on the comparison between HFD and HMT. The top 10 altered functional categories included several lipid metabolic pathways: triacylglycerol biosynthesis, acetyl-coA biosynthesis III (from citrate), palmitate biosynthesis I (animals), and fatty acid biosynthesis initiation II. Thus, we focused on the top altered genes in these categories, which are listed in Table 6.

**Figure 9:**
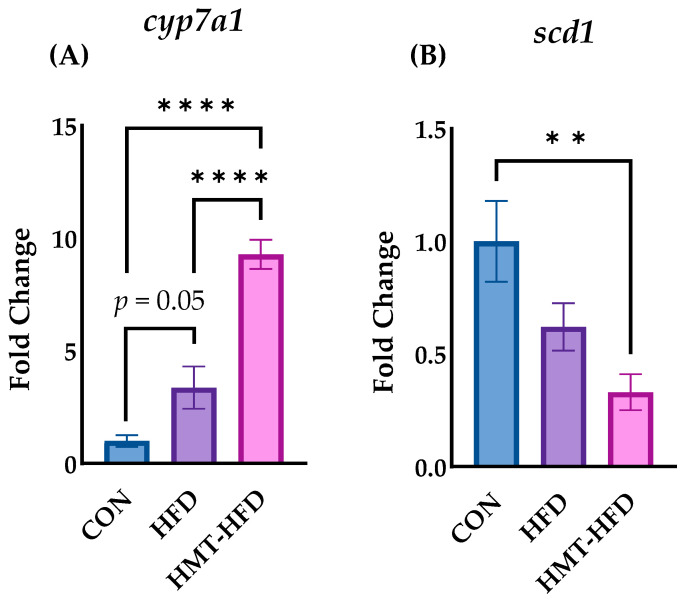
Changes in the hepatic genes (**A**) Cyp7a1 and (**B**) Scd1. Data are shown as means ± standard error of the mean (SEM), *n* = 8 or 9. CON, control diet; HFD, high-fat diet; HMT, heat-moisture treatment. ** *p* < 0.01 and **** *p* < 0.0001 between the indicated groups.

We performed RT-PCR using liver samples for further corroboration, based on the results of biochemical analyses, DNA microarray, and IPA analyses. *Cyp7a1*, a gene that encodes cholesterol 7 α-hydroxylase (CYP7A1), a key enzyme in cholesterol-to-bile acid metabolism, was significantly upregulated in the HMT group compared to in the CON or HFD groups (Figure 9A). *Scd1*, a gene that encodes stearoyl-coenzyme A desaturase 1, the classical rate-limiting enzyme of lipid synthase, was significantly downregulated in the HMT group (Figure 9B).

#### 3.2.5. Microbial Community Composition and Alpha Diversity

**Figure 10:**
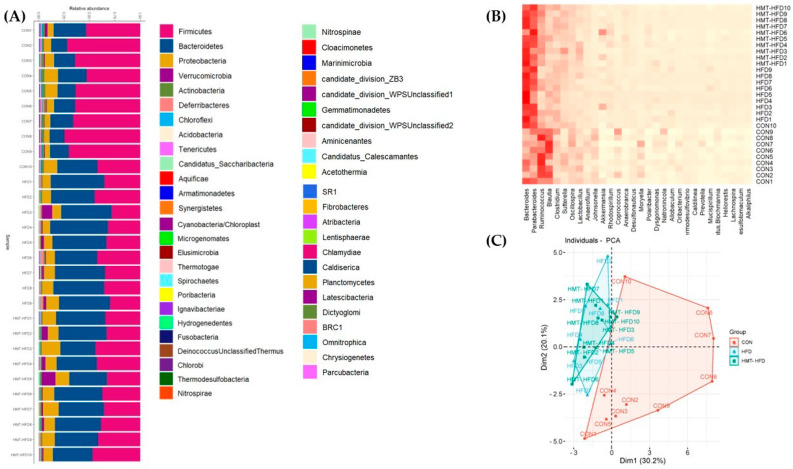

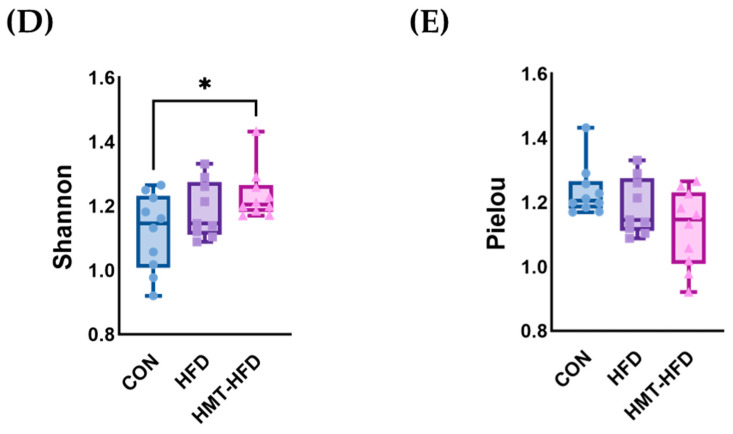
Gut microbial composition in each sample. (**A**) Relative abundance of intestinal microbiota at the genus level; (**B**) Heat map of the primary intestinal microbiota at the genus level; (**C**) Principal component analysis (PCA) plot; (**D**,**E**) Shannon and Pielou indices of each group. CON, control diet; HFD, high-fat diet; HMT, heat−moisture treatment. * *p* < 0.05.

Figure 10A,B show the microbial community composition of each sample at the genus level (the top 30 types). Changes in the gut bacterial composition were summarized, and statistical differences among the three groups were calculated. Figure 9C shows a clear distinction between the CON and HFD/HMT groups. *Bacteroides* are *Parabacteroides* were the most upregulated microbiota through HFD administration, leading to a significant elevation from 11.9 ± 6.5% (CON) to 31.3 ± 7.2% (HFD) and 25.0 ± 5.6% (HMT) for *Bacteroides*, and a significant elevation from 11.9 ± 4.4% (CON) to 21.8 ± 4.6% (HFD) and 21.3 ± 4.1% (HMT) for *Parabacteroides*. Another noteworthy genus is *Blautia*, which was significantly downregulated from 14.7 ± 5.5% (CON) to 6.7 ± 2.2% (HFD) and 5.7 ± 1.8% (HMT), respectively. *Ruminococcus* was 11.4 ± 2.2% in the CON group and decreased to 5.9 ± 3.1% in the HFD group. However, its levels were elevated in the HMT group (17.5 ± 6.4%). Moreover, the HMT group showed area intersection with the HFD group in the principal component analysis (PCA) plot (Figure 10C), indicating that HMT induced divergence of the intestinal microbiota to a certain degree.

Alpha diversity of the bacterial communities was calculated using Shannon index. We found that the Shannon index was significantly higher in the HMT group than that in the CON group. Treatment with HFD decreased alpha diversity indices to some extent compared to the CON group, although the difference was not statistically significant (Figure 10D). No significant difference was found in the Pielou index, indicating that the results are reliable. These data suggest that HFD induced a unique characteristic of gut microbiota; HMT did not manage to restore the balance, although it enhanced the alpha diversity to some extent.

## 4. Discussion

Studies in recent years have frequently reported the health-promoting effects of HA rice flour and HMT starches [15]. The mechanisms involved can increase satiety, improve the glycemic index (mice model, [16]), lower fat absorption and cholesterol levels (human trial, [17]), and alter gut microbiota (rat model, [18]). In the present study, we reproduced some of these reported results. In Study 1, post-harvest HMT, rather than inheritance, contributed to the overall anti-obesity and cholesterol-lowering properties of the rice flour. Based on the results of Study 1, we conducted Study 2, employing HA-HMT rice flour, and found that HA-HMT exhibited the greatest potential for regulating lipid metabolism in the context of HFD.

As predicted, HMT rice flour reversed the weight increase caused by the high-fat component, improving circulating and hepatic lipid metabolism. Liu et al. reported that heat-moisture-treated high-amylose starch (HACs) increased the hepatic expression of *Acat1*, *Cyp8b1*, and *Hmgcoa-R*, while decreasing the hepatic expression of *Ldlr* and *Serbps* in ovariectomized (OVX) rats [18]. Our PCR results indicated that *Cyp7a1* and *Scd1* were candidate genes. CYP7A1 is a rate-limiting enzyme in cholesterol metabolism and is a therapeutic target for cholesterol-lowering [19]. In humans, deficiency of *Cyp7a1* induces a hypercholesterolemic phenotype [20]. In fact, another report by Liu et al. in 2010 reported *Cyp7a1* as a novel gene that prevents hyperlipidemia in OVX rats [21]. However, they also argued that HACs showed no effects on the regulation of genes involved in cholesterol metabolism in the presence of excessive dietary fructose (482 g/kg diet). Our results indicate that if the diet has 45% fat content (Study 2), *Cyp7a1* could be regulated through HMT. *Scd1* is downstream of *Serbp* and is reported to be regulated through HACs [22]; studies have shown that *Scd1* deficiency or disruption protects against hypertriglyceridemia [22]. In addition, our transcriptomic results indicate that *Fasn, Gpt3, Lipin,* and *Fabp5* might also be involved in the mechanism through which HMT contributes to cholesterol-lowering. However, further studies are warranted.

Campesterol is a plant sterol that is structurally similar to cholesterol and is found in many foods, especially vegetable oils, nuts, and seeds [23]. It is absorbed by the human body and can compete with cholesterol for absorption, potentially leading to low blood cholesterol levels [24]. It is often used as a biomarker for dietary intake of plant sterols. In contrast, lathosterol is a cholesterol precursor that is produced in the liver and further converted into cholesterol [25]. It is often used as a biomarker for cholesterol synthesis and is positively correlated with blood cholesterol levels. High levels of lathosterol can indicate increased cholesterol synthesis in the body [26]. In patients with T2D, the plasma level of campesterol is reported to be low [27], whereas a higher lathosterol profile is found [28]. In the present study, we demonstrated that campesterol and lathosterol turnovers also exist in HFD-fed rats, while dietary HMT rice flour attenuated this fluctuation. Besides the cholesterol-regulating effects, our results also indicate that HMT rice flour lowered hepatic TG concentration. Zheng et al. evaluated the effects of HMT high-amylose cornstarch on lipid metabolism in rats fed an HFD. Their results showed that HMT cornstarch significantly improved lipid profiles and reduced hepatic lipid accumulation [12]. They also found that the enzyme activity of alanine aminotransferase and aspartate transaminase was lower in the HMT group with HFD compared to that in the HFD group. Our results of IPA analyses indicated that genes, including fatty acid synthase (*Fasn*), glycerol-3-phosphate O-acyltransferase 3 (*Gpat3*), and lipin 1 (*Lpin1*), may also contribute to the lipid-regulating effects. Hence, future studies should focus on investigating these genes.

Several studies have reported that a high amylose or RS content regulates gut microbial communities and microbial metabolites [4,29]. In HFD-fed mice, high-amylose corn starch increased and decreased the relative abundances of *Firmicutes* and *Bacteroidetes*, respectively, and showed similar regulating properties as inulin, a well-studied prebiotic [30]. High-amylose maize increased the alpha diversity in HFD-fed mice and restored the populations of *Thiotrichaceae, Bifidobacteriaceae, Streptococcaceae, Enterococcaceae, Thermoanaerobacteraceae, Deferribacteraceae, Porphyromonadaceae, Muribaculaceae, Barnesiellaceae, Lactobacillaceae,* and *Mogibacteriaceae* [31]. In HFD-fed Kunming mice, RS administration restored the relative abundances of *Bacteroides*, *Ruminococcus,* and *Bifidobacterium* [32]. In the present study, we found increased alpha diversity at the genus level, and the relative abundance of *Ruminococcus* was enhanced through HMT. Previous reports have suggested that *Ruminococcus* plays a key role in the degradation of dietary RS into small molecular fragments for further digestion [33]. Because our HMT group’s diet contained RS, the unique increase in the *Ruminococcus* taxa in the HMT group might be a consequence of the RS component. The differentially regulated microbial profiles in our study might be attributable to the amylose content and animal species; therefore, further studies are required to reveal how HMT rice flour affects the human gut microbiome.

In conclusion, our findings indicate that the HMT, rather than the inheritance of HA rice, is responsible for the observed anti-obesity and cholesterol-lowering effects. We identified *Cyp7a1* and *Scd1* as candidate genes involved in the cholesterol-regulating potential of HMT rice flour, and highlighted the roles of *Fasn, Gpt3, Lipin*, and *Fabp5* in this process. Furthermore, our study has shown that HMT rice flour can influence the gut microbiome, particularly the *Ruminococcus* taxa, which is likely attributable to the RS component of the rice flour. However, further research is needed to elucidate the precise mechanisms underlying these effects and to establish their relevance to human gut microbiome and health.

## Figures and Tables

**Table 1:** Nutrition information of diets used in Study 1.

Weight/Calories g (kcal)	LA-nHMT	LA-HMT	HA-nHMT	HA-HMT
Casein	24.4 (97.6)	24.4 (97.6)	24.4 (97.6)	24.4 (97.6)
D, L-methionine	0.3 (-)	0.3 (-)	0.3 (-)	0.3 (-)
α-cornstarch	41.2 (164.8)	-	-	-
Rice flour	-	34.7 (140.5)	34.3 (142.8)	34.3 (142.8)
Carbohydrate	-	31.8 (127.1)	30.4 (121.6)	30.4 (121.6)
Protein	-	2.5 (10.1)	2.8 (11.2)	2.8 (11.2)
Fat	-	0.4 (3.3)	1.1 (10.0)	1.1 (10.0)
Corn oil	1 (9)	1 (9)	1 (9)	1 (9)
Cellulose	5 (-)	5 (-)	5 (-)	5 (-)
AIN-76 mineral mix	1 (-)	1 (-)	1 (-)	1 (-)
AIN-76 vitamin mix	3.5 (-)	3.5 (-)	3.5 (-)	3.5 (-)
Lard	23.4 (210.6)	23.4 (210.6)	23.4 (210.6)	23.4 (210.6)
Sucrose	0.2 (0.8)	0.2 (0.8)	0.2 (0.8)	0.2 (0.8)
Total	100 (482.8)	100 (458.5)	100 (460.8)	100 (460.8)

Note: The values are presented as g (kcal), [gram (kilocalorie)]. Carbohydrate, protein, and fat indicate their ratios in the added rice flour. LA, low-amylose; HA, high-amylose; nHMT, without heat-moisture treatment; HMT, heat-moisture treatment; HFD, high-fat diet; LA-nHMT, low-amylose, high-fat diet without HMT; LA-HMT, low-amylose, high-fat diet with HMT; HA-nHMT, high-amylose, high-fat diet without HMT; HA-HMT, high-amylose, high-fat diet with HMT.

**Table 2:** Nutrition information of diets used in Study 2.

Weight/Calories g (kcal)	CON	HFD	HMT
Casein	18.5 (74)	23.3 (93.2)	23.0 (92)
D, L-methionine	0.3 (-)	0.3 (-)	0.3 (-)
Rice flour	66.5 (249.4)	43.3 (162.5)	44 (161.4)
Carbohydrate	58.9 (235.4)	38.3 (153.3)	35.3 (141.2)
Protein	3.1 (12.2)	2 (8)	2.7 (10.7)
Fat	0.2 (1.8)	0.13 (1.2)	1.1 (9.5)
Corn oil	5 (45)	1 (9)	1 (9)
Cellulose	5 (-)	5 (-)	5 (-)
AIN-76 mineral mix	1 (-)	1 (-)	1 (-)
AIN-76 vitamin mix	3.5 (-)	3.5 (-)	3.5 (-)
Lard	-	22.4 (201.6)	22 (198)
Sucrose	0.2 (0.8)	0.2 (0.8)	0.2 (0.8)
Total	100 (369.2)	100 (467.0)	100 (460.8)
Cholesterol	-	0.25 (-)	0.25 (-)

Note: the values are presented as g (kcal), [gram (kilocalorie)]. Carbohydrate, protein, and fat indicate their ratios in the added rice flour. HMT, heat-moisture treatment; CON, control diet, without HMT; HFD, high-amylose, high-fat diet without HMT; HMT, high-amylose, high-fat diet with HMT; CHO, cholesterol.

**Table 3:** Organ and tissue weights, and blood and tissue biochemical parameters in Study 1.

Organ or Tissue/Biochemical Parameters	LA-nHMT	LA-HMT	HA-nHMT	HA-HMT	Main Effect, nHMT vs. HMT	Main Effect, LA vs. HA	Interaction Effect
Liver, g	11.8 ± 1.4	11.2 ± 1.3	10.6 ± 0.8	10.6 ± 1.2	*p* = 0.06	n.s.	n.s.
Total adipose tissue, g	35.2 ± 4.9	32.3 ± 5.5	34.1 ± 9.5	27.6 ± 2.2	*p* = 0.07	n.s.	n.s.
Retroperitoneal fat pad	11.5 ± 3.3	11.3 ± 2.5	11.9 ± 3.6	9.2 ± 1.4	n.s.	n.s.	n.s.
Mesenteric fat pad	7.4 ± 1.5	7.2 ± 1.0	8.1 ± 2.1	6.4 ± 0.9	n.s.	n.s.	n.s.
Epididymal fat pad	13.8 ± 3.9	13.8 ± 2.8	14.2 ± 4.1	11.4 ± 0.9	n.s.	n.s.	n.s.
Total skeletal muscle tissue, g	5.1 ± 0.6	4.9 ± 0.5	4.7 ± 0.3	4.8 ± 0.3	n.s.	n.s.	n.s.
Gastrocnemius muscle	4.5 ± 0.7	4.3 ± 0.5	4.1 ± 0.6	4.1 ± 0.3	n.s.	n.s.	n.s.
Soleus muscle	0.65 ± 0.1	0.60 ± 0.1	0.65 ± 0.4	0.65 ± 0.1	n.s.	n.s.	n.s.
BW, g	478.9 ± 27.3	474.1 ± 29.9	463.2 ± 27.5	450.3 ± 17.5	*	n.s.	n.s.
Liver/BW, %	2.5 ± 0.2	2.3 ± 0.1	2.3 ± 0.1	2.4 ± 0.2	n.s.	n.s.	n.s.
Adipose/BW, %	7.2 ± 0.9	6.8 ± 0.9	7.3 ± 1.7	6.1 ± 0.5	*p* = 0.07	n.s.	n.s.
Skeletal Muscle/BW, %	1.1 ± 0.1	1.0 ± 0.1	1.0 ± 0.1	1.1 ± 0.1	*p* = 0.07	n.s.	n.s.
Plasma, TG (mg/dL)	66.1 ± 25.3	53.0 ± 17.8	61.1 ± 23.8	69.7 ± 24.5	n.s.	n.s.	n.s.
Plasma, T-Cho (mg/dL)	56.2 ± 8.3	62.5 ± 11.0	60.4 ± 16.2	52.5 ± 12.7	n.s.	n.s.	n.s.
Plasma, HDL-Cho (mg/dL)	38.6 ± 4.2	50.7 ± 8.5	50.8 ± 23.6	45.3 ± 19.9	n.s.	n.s.	n.s.
Plasma, LDL-Cho (mg/dL)	17.1 ± 8.0	11.7 ± 11.3	13.2 ± 7.8	7.23 ± 10.4	n.s.	n.s.	n.s.
Liver, TG (mg/g)	70.7 ± 14.7	66.2 ± 6.4	51.8 ± 9.8	62.5 ± 9.1	n.s.	*	n.s.
Liver, T-Cho (mg/g)	13.8 ± 2.0	12.0 ± 1.4	12.6 ± 1.8	11.7 ± 0.4	*	n.s.	n.s.

Data are shown as the mean ± standard error of the mean (SEM), *n* = 6–8. LA, low-amylose; HA, high-amylose; nHMT, without heat-moisture treatment; HMT, heat-moisture treatment; LA-nHMT, low-amylose, without HMT; LA-HMT, low-amylose, with HMT; HA-nHMT, high-amylose, without HMT; HA-HMT, high-amylose, with HMT; BW, body weight; TG, triglyceride; T-Cho, total cholesterol; LDL-Cho, low-density lipoprotein cholesterol; HDL-Cho, high-density lipoprotein cholesterol; n.s., no significance. * *p* < 0.05.

**Table 4:** Final body weight, and organ and tissue masses in Study 2.

Organ or Tissue	CON	HFD	HMT
Liver, g	14.9 ± 2.1	20.5 ± 2.3 *	17.2 ± 2.9
Total adipose tissue, g	30.4 ± 6.5	40.2 ± 10.4 *	34.2 ± 5.3
Retroperitoneal fat pad	6.3 ± 1.5	8.1 ± 2.2	6.8 ± 1.5
Mesenteric fat pad	13.0 ± 2.9	16.9 ± 5.4	14.4 ± 2.5
Epididymal fat pad	11.2 ± 2.9	15.1 ± 3.5 *	12.0 ± 2.9
Total skeletal muscle tissue, g	5.8 ± 0.6	6.2 ± 0.5	5.5 ± 0.5
Gastrocnemius muscle	5.4 ± 0.6	5.8 ± 0.5	5.2 ± 0.5
Soleus muscle	0.4 ± 0.3	0.4 ± 0.1	0.4 ± 0.1
BW, g	464.8 ± 44.9	524.3 ± 57.9 *^#^	468.7 ± 37.5
Liver/BW, %	3.2 ± 0.2	3.9 ± 0.2 ***	3.6 ± 0.4 **
Adipose/BW, %	6.5 ± 1.1	7.6 ± 1.2 (*p* = 0.05, vs. CON)	7.2
Skeletal Muscle/BW, %	1.3 ± 0.1	1.2 ± 0.1	1.2 ± 0.1

Data are shown as the mean ± standard error of the mean (SEM), *n* = 8–10. CON, control diet; HFD, high-fat diet; HMT, heat-moisture treatment; BW, final body weight. * *p* < 0.05, ** *p* < 0.01, and *** *p* < 0.001 vs. CON. ^#^ *p* < 0.05 vs. HFD.

**Table 5:** Campesterol/cholesterol and lathosterol/cholesterol profiles revealed via metabolomic analyses.

Metabolites	HFD vs. CON		HMT vs. CON		HMT vs. HFD	
	Ratio	*p*-Value	Ratio	*p*-Value	Ratio	*p*-Value
**Campesterol/Cholesterol** **Lathosterol/** **Cholesterol**	0.2 2.9	0.006 < 0.001	0.4 2.4	0.019 0.140	1.9 0.8	0.004 0.430

CON, control diet; HFD, high-fat diet; HMT, heat-moisture treatment.

**Table 6:** Top differentially expressed genes in the functional categories characterized via IPA.

Probe	Gene	Log Ratio
*Fasn*	fatty acid synthase	−1.62
*Gpat3*	Glycerol-3-phosphate O-acyltransferase 3	−0.92
*Lpin1*	lipin1	−0.90
*Fabp5*	fatty acid binding protein 5, epidermal	−0.81
*Aldh1b1*	aldehyde dehydrogenase 1 family, member B1	−0.77
*Scd1*	Stearoyl-Coenzyme A desaturase	−0.71
*Acly*	ATP citrate lyase	−0.70
*Cd14*	CD14 molecule	0.59
*Fmo5*	flavin containing monooxygenase 5	−0.36

## Data Availability

All microarray data are MIAME compliant and have been deposited in a MIAME compliant database, the National Center for Biotechnology Information (NCBI) Gene Expression Omnibus (http://www.ncbi.nlm.nih.gov/geo/, accessed on 3 April 2023, GEO Series accession number GSE226305, https://www.ncbi.nlm.nih.gov/geo/query/acc.cgi?acc=GSE226305 (accessed on 3 April 2023).

## References

[1] Wittwer J.A., Golden S.H., Joseph J.J. (2020). Diabetes and CVD Risk: Special Considerations in African Americans Related to Care. Curr. Cardiovasc. Risk Rep..

[2] Neuhouser M.L. (2019). The Importance of Healthy Dietary Patterns in Chronic Disease Prevention. Nutr. Res..

[3] Tian S., Sun Y. (2020). Influencing Factor of Resistant Starch Formation and Application in Cereal Products: A Review. Int. J. Biol. Macromol..

[4] DeMartino P., Cockburn D.W. (2020). Resistant Starch: Impact on the Gut Microbiome and Health. Curr. Opin. Biotechnol..

[5] Dobranowski P.A., Stintzi A. (2021). Resistant Starch, Microbiome, and Precision Modulation. Gut Microbes.

[6] Snelson M., Kellow N.J., Coughlan M.T. (2019). Modulation of the Gut Microbiota by Resistant Starch as a Treatment of Chronic Kidney Diseases: Evidence of Efficacy and Mechanistic Insights. Adv. Nutr..

[7] Di Rosa C., De Arcangelis E., Vitelli V., Crucillà S., Angelicola M., Trivisonno M.C., Khazrai Y.M. (2023). Effect of Three Bakery Products Formulated with High-Amylose Wheat Flour on Post-Prandial Glycaemia in Healthy Volunteers. Foods.

[8] Gu F., Li C., Hamaker B.R., Gilbert R.G., Zhang X. (2020). Fecal Microbiota Responses to Rice RS3 Are Specific to Amylose Molecular Structure. Carbohydr. Polym..

[9] Wu F., Yang N., Touré A., Jin Z., Xu X. (2013). Germinated Brown Rice and Its Role in Human Health. Crit. Rev. Food Sci. Nutr..

[10] da Rosa Zavareze E., Dias A.R.G. (2011). Impact of Heat-Moisture Treatment and Annealing in Starches: A Review. Carbohydr. Polym..

[11] Van Hung P., Vien N.L., Phi N.T.L. (2016). Resistant Starch Improvement of Rice Starches under a Combination of Acid and Heat-Moisture Treatments. Food Chem..

[12] Zheng B., Wang H., Shang W., Xie F., Li X., Chen L., Zhou Z. (2018). Understanding the Digestibility and Nutritional Functions of Rice Starch Subjected to Heat-Moisture Treatment. J. Funct. Foods.

[13] da Rosa Zavareze E., Storck C.R., de Castro L.A.S., Schirmer M.A., Dias A.R.G. (2010). Effect of Heat-Moisture Treatment on Rice Starch of Varying Amylose Content. Food Chem..

[14] Mopuri R., Kalyesubula M., Rosov A., Edery N., Moallem U., Dvir H. (2021). Improved Folch method for liver-fat quantification. Front. Vet. Sci..

[15] Sui Z., Yao T., Ye X., Bao J., Kong X., Wu Y. (2017). Physicochemical Properties and Starch Digestibility of In-Kernel Heat-Moisture-Treated Waxy, Low-, and High-Amylose Rice Starch. Starch-Stärke.

[16] Lee C.J., Kim Y., Choi S.J., Moon T.W. (2012). Slowly digestible starch from heat-moisture treated waxy potato starch: Preparation, structural characteristics, and glucose response in mice. Food Chem..

[17] Yagi M., Takabe W., Wickramasinghe U., Okuda F., Kom M., Fujimura A., Yonei Y. (2018). Effect of Heat-Moisture-Treated High-Amylose Corn Starch-Containing Food on Postprandial Blood Glucose. Glycative Stress Res..

[18] Wu T.Y., Tsai S.J., Sun N.N., Dai F.J., Yu P.H., Chen Y.C., Chau C.F. (2020). Enhanced Thermal Stability of Green Banana Starch by Heat-Moisture Treatment and Its Ability to Reduce Body Fat Accumulation and Modulate Gut Microbiota. Int. J. Biol. Macromol..

[19] Liu X., Ogawa H., Ando R., Nakakuki T., Kishida T., Ebihara K. (2007). Heat-Moisture Treatment of High-Amylose Corn Starch Increases Dietary Fiber Content and Lowers Plasma Cholesterol in Ovariectomized Rats. J. Food Sci..

[20] Chambers K.F., Day P.E., Aboufarrag H.T., Kroon P.A. (2019). Polyphenol Effects on Cholesterol Metabolism via Bile Acid Biosynthesis, CYP7A1: A Review. Nutrients.

[21] Pullinger C.R., Eng C., Salen G., Shefer S., Batta A.K., Erickson S.K., Kane J.P. (2002). Human Cholesterol 7α-Hydroxylase (CYP7A1) Deficiency Has a Hypercholesterolemic Phenotype. J. Clin. Investig..

[22] Liu X., Ogawa H., Kishida T., Ebihara K. (2010). The Effect of High-Amylose Cornstarch on Lipid Metabolism in OVX Rats Is Affected by Fructose Feeding. J. Nutr. Biochem..

[23] Li M., Wang J., Wang F., Strappe P., Liu W., Zheng J., Zhang Y. (2021). Microbiota Fermentation Characteristics of Acylated Starches and the Regulation Mechanism of Short-Chain Fatty Acids on Hepatic Steatosis. Food Funct..

[24] Chu K., Miyazaki M., Man W.C., Ntambi J.M. (2006). Stearoyl-Coenzyme A Desaturase 1 Deficiency Protects against Hypertriglyceridemia and Increases Plasma High-Density Lipoprotein Cholesterol Induced by Liver X Receptor Activation. Mol. Cell. Biol..

[25] Jaceldo-Siegl K., Lütjohann D., Sirirat R., Mashchak A., Fraser G.E., Haddad E. (2017). Variations in Dietary Intake and Plasma Concentrations of Plant Sterols across Plant-Based Diets among North American Adults. Mol. Nutr. Food Res..

[26] Jojima T., Sakurai S., Wakamatsu S., Iijima T., Saito M., Tomaru T., Aso Y. (2021). Empagliflozin Increases Plasma Levels of Campesterol, a Marker of Cholesterol Absorption, in Patients with Type 2 Diabetes: Association with a Slight Increase in High-Density Lipoprotein Cholesterol. Int. J. Cardiol..

[27] Kempen H., Glatz J.F., Leuven J.G., van der Voort H.A., Katan M.B. (1988). Serum Lathosterol Concentration Is an Indicator of Whole-Body Cholesterol Synthesis in Humans. J. Lipid Res..

[28] Matsumura T., Ishigaki Y., Nakagami T., Akiyama Y., Ishibashi Y., Ishida T., Shoji T. (2022). Relationship between Diabetes Mellitus and Serum Lathosterol and Campesterol Levels: The CACHE Study DM Analysis. J. Atheroscler. Thromb..

[29] Nielsen T.S., Bendiks Z., Thomsen B., Wright M.E., Theil P.K., Scherer B.L., Marco M.L. (2019). High-Amylose Maize, Potato, and Butyrylated Starch Modulate Large Intestinal Fermentation, Microbial Composition, and Oncogenic miRNA Expression in Rats Fed a High-Protein Meat Diet. Int. J. Mol. Sci..

[30] Hu J., Zheng P., Qiu J., Chen Q., Zeng S., Zhang Y., Zheng B. (2022). High-amylose corn starch regulated gut microbiota and serum bile acids in high-fat diet-induced obese mice. Int. J. Mol. Sci..

[31] Xie Z., Yao M., Castro-Mejía J.L., Ma M., Zhu Y., Fu X., Zhang B. (2023). Propionylated high-amylose maize starch alleviates obesity by modulating gut microbiota in high-fat diet-fed mice. J. Funct. Foods.

[32] Chen X., Wang Z., Wang D., Kan J. (2023). Effects of resistant starch III on the serum lipid levels and gut microbiota of Kunming mice under high-fat diet. Food Sci. Hum. Wellness.

[33] Ze X., Duncan S.H., Louis P., Flint H.J. (2012). Ruminococcus bromii is a keystone species for the degradation of resistant starch in the human colon. ISME J..

